# Massive pericardial effusion due to chronic active Epstein–Barr virus infection successfully treated with PD-1 blockade: A case report

**DOI:** 10.1097/MD.0000000000030298

**Published:** 2022-08-26

**Authors:** Yubo Pi, Jingshi Wang, Zhao Wang

**Affiliations:** a Department of Hematology, Beijing Friendship Hospital, Capital Medical University, Beijing, China; b Department of Hematology, Beijing Friendship Hospital, Capital Medical University, Beijing, China; c Department of Hematology, Beijing Friendship Hospital, Capital Medical University, Beijing, China.

**Keywords:** case report, chronic active Epstein–Barr virus infection, massive pericardial effusion, PD-1 blockade

## Abstract

Chronic active Epstein–Barr virus (EBV) infection (CAEBV) is a rare but life-threatening EBV-positive lymphoproliferative disorder. Currently, treatment options for CAEBV are limited. Allogeneic hematopoietic stem cell transplantation (allo-HSCT) is the only way to cure CAEBV. Here, we report a rare case of CAEBV manifesting as massive pericardial effusion that was successfully treated with programmed cell death protein-1 (PD-1) blockade immunotherapy.

**Patient concerns:**

A 36-year-old woman with intermittent chest distress and dyspnea for 8 months was admitted to our center on October 25, 2021. Laboratory tests showed leukocytopenia and elevated liver enzyme levels. Initial echocardiography revealed massive pericardial effusion.

**Diagnosis:**

High levels of EBV-DNA were detected in the pericardial fluid by metagenomic next-generation sequencing. The pathological diagnosis of her left inguinal lymph node and skin lesions revealed systemic CAEBV.

**Interventions:**

The patient received sintilimab injection at a dose of 200 mg every 2 weeks in combined with lenalidomide 10 mg once daily.

**Outcomes:**

The patient achieved complete resolution of pericardial effusion 5 months after PD-1 blockade immunotherapy without apparent adverse effects.

**Lessons:**

CAEBV is a rare but life-threatening EBV-positive lymphoproliferative disease. We present a rare case of massive pericardial effusion caused by systemic CAEBV, which was successfully treated with sintilimab. This case highlights the promising curative effect of PD-1 blockade immunotherapy in systemic CAEBV, especially for patients not suitable for allo-HSCT.

## 1. Introduction

Chronic active Epstein–Barr virus infection (CAEBV) is a rare and life-threatening condition characterized by high levels of Epstein–Barr virus (EBV)-DNA in the peripheral blood and abnormal lymphoproliferation of EBV-positive T- or natural killer (NK) cells in infected tissues. It is most frequent in Asians and Native Americans from Central and South America.^[1,2]^ Although CAEBV often develops in children and adolescents, adult-onset CAEBV has been reported. Systemic CAEBV often presents with persistent or recurrent infectious mononucleosis (IM)-like symptoms, including fever, cytopenia, liver insufficiency, hepatomegaly, and splenomegaly.^[3]^ Pericardial effusion was also described in 20% of patients according to a small-sample study in Japan.^[4]^

Without proper treatment, CAEBV can progress to EBV-associated hemophagocytic lymphohistiocytosis (HLH), lymphoma, and multiple organ failure. Prognosis is associated with EBV-DNA viral loads, platelet levels, and age at onset.^[5,6]^ At present, treatment options for patients with CAEBV are limited. Allogeneic hematopoietic stem cell transplantation (allo-HSCT) is the only way to cure CAEBV; however, severe complications due to EBV infection, lack of appropriate donors, and disease relapse after transplantation remain challenging. Interestingly, it has been reported that programmed cell death protein-1 (PD-1) is upregulated on the surface of antigen-activated T cells in chronic viral infections and that PD-1 blockade restores T-cell immune function in EBV-associated HLH.^[7,8]^

Here, we report a rare case of CAEBV manifesting as massive pericardial effusion that was successfully treated with PD-1 blockade immunotherapy.

## 2. Case report

A 36-year-old woman with intermittent chest distress and dyspnea for 8 months was admitted to our center on October 25, 2021. Approximately 8 months previously, the patient was admitted to a local hospital because of chest distress and dyspnea. Laboratory tests revealed leukocytopenia and elevated liver enzyme levels. Echocardiography revealed a massive pericardial effusion. She was treated with percutaneous pericardial drainage, and the pericardial fluid was exudative according to Light’s criteria. She had intermittent chest distress and dyspnea ever since and underwent bimonthly percutaneous pericardial drainage. The patient was previously healthy. After admission, the patient’s temperature was 36°C, heart rate was 88 bpm, respiratory rate was 20 bpm, blood pressure was 105/72 mm Hg, and oxygen saturation in room air was 98%. Physical examination revealed facial skin rashes, low- and dull-heart sounds, and multiple superficial lymph node enlargement.

**Figure 1.**
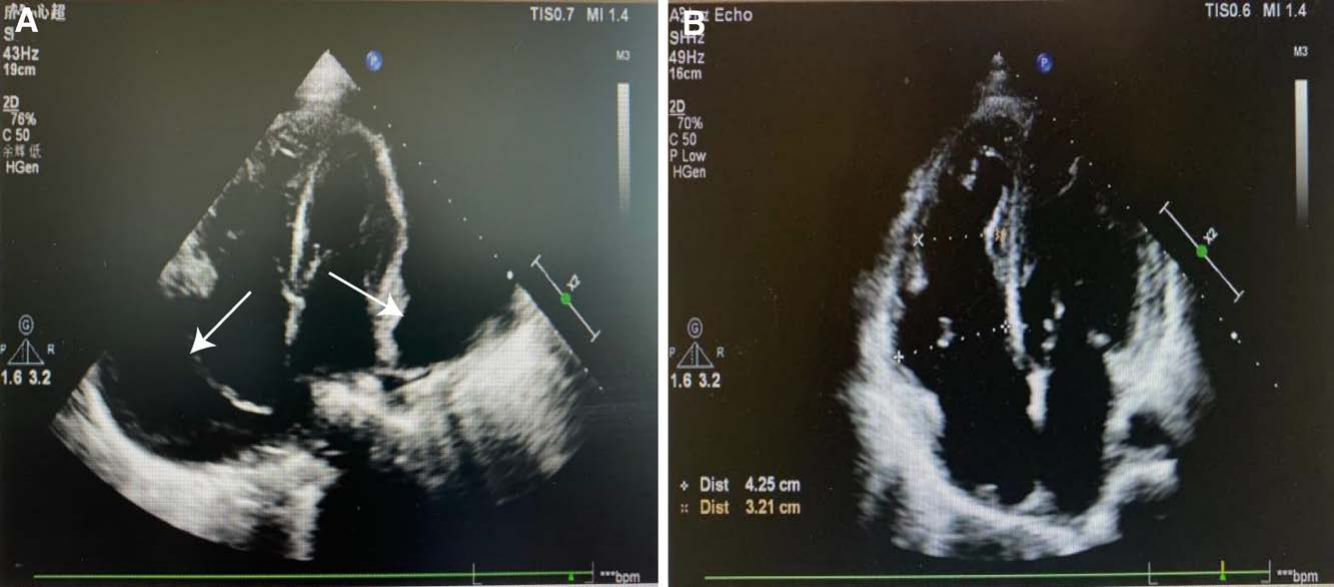
Echocardiography before and 5 mo after sintilimab administration. (A) An initial echocardiography revealed massive pericardial effusion (white arrow). (B) A follow-up echocardiography 5 mo after sintilimab administration showed that the patient’s pericardial effusion was completely absorbed.

Blood analysis revealed leukocytopenia (3.09 × 10^9^/L, normal range 3.5–9.5 × 10^9^/L), elevated liver enzymes (alanine aminotransferase 44 U/L, normal range 7–40 U/L; aspartate aminotransferase 102.4 U/L, normal range 13–35 U/L), and increased erythrocyte sedimentation rate (33 mm/hr; normal range 0–20 mm/hr). Electrocardiography showed ectopic atrial rhythm, atrial tachycardia with the Wenckebach phenomenon, paired atrial premature beats, and first-degree atrioventricular block. Initial echocardiography revealed a massive pericardial effusion with a pericardial separation of 5.03 cm (Fig. 1A). Ultrasonography confirmed enlargement of multiple superficial lymph nodes. Full-body computed tomography showed soft tissue swelling of the head and neck, massive pericardial effusion, pulmonary interstitial edema, and mild hepatosplenomegaly. Following administration of contrast media, no signs of malignancy were noted.

Pericardiocentesis was performed, and the pericardial fluid was exudative according to Light’s criteria. Cytology and flow cytometric analysis indicated a lymphocyte proportion of 92.26%, predominantly mature T cells. Metagenomic next-generation sequencing revealed high levels of EBV-DNA in the pericardial fluid (6.3 × 10^7^ copies/mL; normal range < 5.0 × 10^2^ copies/mL). The patient was further evaluated for systemic CAEBV. EBV-DNA levels in the patient’s peripheral blood mononuclear cells and plasma were 7.6 × 10^5^ copies/mL and 6.6 × 10^3^ copies/mL, respectively, involving T- and NK-cell lineages. Pathological examination of skin lesions indicated infiltration of abnormal T cells, of which immunohistochemical staining was positive for CD3, CD5 (sporadic), CD4 (sporadic), CD2, CD7, CD8, GrB, and TIA-1, and negative for CD20 and CD56. Pathological examination of her left inguinal lymph node also showed infiltration of abnormal T cells, of which immunohistochemical staining was positive for CD3, CD20 (sporadic), CD2, and CD5, and negative for CD56, TIA-1, and GrB. EBV-encoded RNA (EBER), examined by in situ hybridization, was positive in both the skin lesions and left inguinal lymph nodes. There was no evidence of underlying immunodeficiency. The final diagnosis was systemic CAEBV.

**Figure 2.**
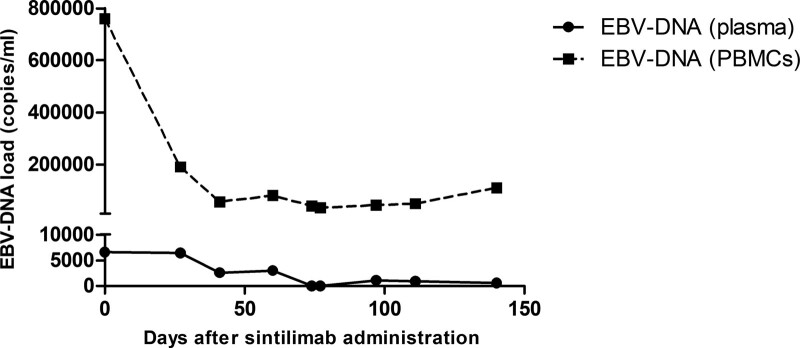
Changes in EBV-DNA levels in the patient’s PBMCs and plasma throughout treatment. EBV = Epstein–Barr virus, PBMC = peripheral blood mononuclear cell.

The patient’s heart was in critical condition due to massive pericardial effusion and various forms of arrhythmia, which deprived her of the possibility of chemotherapies and allo-HSCT. Therefore, sintilimab (a PD-1 monoclonal antibody) was administered at a dose of 200 mg every 2 weeks in combined with lenalidomide 10 mg once daily. The patient achieved complete resolution of pericardial effusion 5 months after PD-1 blockade immunotherapy (Fig. 1B), and the EBV-DNA levels in the patient’s peripheral blood mononuclear cells and plasma were also decreased (Fig. 2).

## 3. Discussion

EBV, a human gamma herpesvirus, infects >90% of the population worldwide. While childhood EBV infection is ordinarily asymptomatic, adolescent EBV infection often manifests as self-limiting IM. In rare cases, it can also result in CAEBV. CAEBV is a rare but life-threatening EBV-positive lymphoproliferative disease characterized by high levels of EBV in the blood and tissues and organ infiltration of EBV-infected T- and NK cells.^[1,2,5]^ Currently, the diagnosis of CAEBV should meet the following criteria: IM-like symptoms persisting for >3 months, increased EBV-DNA in the peripheral blood, demonstration of EBV-RNA or viral proteins in affected tissues, and no evidence of immunodeficiency.^[1]^ Most patients with CAEBV have systemic presentations such as fever, hepatosplenomegaly, lymphadenopathy, cytopenia, and skin rash.^[2]^ However, cardio- and vasculotropic infiltration of EBV-infected T- and NK cells leading to cardiac complications has also been documented in patients with CAEBV, with a median time of 3.4 years from disease onset to diagnosis of cardiac complications.^[4]^ However, massive pericardial effusion as a primary manifestation of CAEBV is extremely rare. The prognosis of patients with CAEBV is poor. Without proper treatment, the 3-year overall survival (OS) rate is only 16.7%.^[9]^

Currently, there is no standard treatment for CAEBV, as it is often refractory to antiviral therapy, immunosuppressive agents, intravenous immunoglobulins, and conventional chemotherapy.^[6,9–12]^ Although allogeneic hematopoietic stem cell transplantation (allo-HSCT) remains the only curative treatment, the mortality rate remains high due to disease relapse and transplantation-related complications.^[5]^ A 3-step therapy was proposed to suppress disease activity before planned allo-HSCT, resulting in a 3-year OS rate of 87.3%.^[9]^ A recent retrospective study in China also demonstrated a favorable 3-year OS rate of 92.0% for patients with CAEBV who received the L-DEP regimen (pegylated-asparaginase, liposomal doxorubicin, etoposide, and methylprednisolone) prior to planned haploidentical HSCT.^[13]^ In addition, JAK inhibitors can also achieve long-term stable remission and provide an opportunity for allo-HSCT.^[11]^ However, cardiac complications in CAEBV patients are beyond cure, and 78% die of disease progression or transplantation-related events.^[4]^ There was a case of pericardial effusion successfully treated with allo-HSCT without severe complications;^[14]^ however, our patient also experienced various forms of arrhythmia indicating possible myocarditis, which occurred in 6% of CAEBV cases and was highly associated with death.^[4]^

PD-1 is expressed on the surface of antigen-activated T cells during chronic viral infections.^[7]^ It has been reported that interactions between PD-1 and PD-L1 can transmit inhibitory signals into antigen-activated T cells. Thus, PD-1 blockade can restore T-cell immune function. According to a recent study, nivolumab could clear EBV infection in 4 out of 7 relapsed or refractory HLH patients and restore anti-EBV function in CD8^+^ T cells.^[8]^ Considering all these factors, the patient agreed to undergo PD-1 blockade immunotherapy. Sintilimab, a PD-1 monoclonal antibody, was administered at a dose of 200 mg every 2 weeks. In an attempt to maximize effectiveness, anti-PD-1 antibodies are often used in combination with chemotherapy, radiotherapy, lenalidomide, Bruton’s tyrosine kinase inhibitor, and chimeric antigen receptor T-cell therapy. Lenalidomide, an immunomodulatory drug, has been reported to have a synergistic effect with anti-PD-1 antibodies.^[15]^ Therefore, lenalidomide 10 mg once daily was also administered. As a result, the patient achieved complete resolution of pericardial effusion 5 months after PD-1 blockade immunotherapy without apparent side effects. Currently, the patient is reluctant to consider allo-HSCT and continues treatment with sintilimab at 3-week intervals. Based on our limited experience, we suggest that the patient should continue treatment with PD-1 blockade immunotherapy at gradually increasing intervals for life unless allo-HSCT is available. In case of disease progression during treatment, allo-HSCT is recommended. However, randomized controlled clinical trials are needed to further explore the efficacy and duration of treatment, possible adverse effects, and timing of allo-HSCT in CAEBV patients with cardiac complications.

In conclusion, CAEBV is a rare but life-threatening EBV-positive lymphoproliferative disease with a poor prognosis. Currently, allo-HSCT is the only curative treatment available. However, CAEBV patients with cardiac complications often die of disease progression or transplantation-related events. We present a rare case of massive pericardial effusion as a primary manifestation of CAEBV, which was successfully treated with sintilimab. This case showed promising curative effect of PD-1 blockade immunotherapy for CAEBV patients with cardiac complications.

## Acknowledgments

We thank all authors for their contribution in this work.

## Author contributions

Writing—original draft: Yubo Pi.

Writing—review & editing: Jingshi Wang, Zhao Wang.

Supervision: Jingshi Wang, Zhao Wang.

## References

[1] Kimura H Ito Y Kawabe S. EBV-associated T/NK–cell lymphoproliferative diseases in nonimmunocompromised hosts: prospective analysis of 108 cases. Blood. 2012;119:673–86. 22096243 10.1182/blood-2011-10-381921

[2] Cohen JI Kimura H Nakamura S. Epstein-Barr virus-associated lymphoproliferative disease in non-immunocompromised hosts: a status report and summary of an international meeting, 8-9 September 2008. Ann Oncol. 2009;20:1472–82. 19515747 10.1093/annonc/mdp064 PMC2731018

[3] Arai A. Advances in the study of chronic active epstein-barr virus infection: clinical features under the 2016 who classification and mechanisms of development. Front Pediatr. 2019;7:14. 30805320 10.3389/fped.2019.00014 PMC6370717

[4] Muneuchi J Ohga S Ishimura M. Cardiovascular complications associated with chronic active Epstein-Barr virus infection. Pediatr Cardiol. 2009;30:274–81. 19184184 10.1007/s00246-008-9343-8

[5] Davila Saldana BJ John T Bonifant C. High risk of relapsed disease in patients with NK/T-cell chronic active Epstein-Barr virus disease outside of Asia. Blood Adv. 2022;6:452–9. 34670275 10.1182/bloodadvances.2021005291 PMC8791566

[6] Yonese I Sakashita C Imadome KI. Nationwide survey of systemic chronic active EBV infection in Japan in accordance with the new WHO classification. Blood Adv. 2020;4:2918–26. 32598475 10.1182/bloodadvances.2020001451 PMC7362364

[7] Xu-Monette ZY Zhang M Li J. PD-1/PD-L1 blockade: have we found the key to unleash the antitumor immune response?. Front Immunol. 2017;8:1597. 29255458 10.3389/fimmu.2017.01597 PMC5723106

[8] Liu P Pan X Chen C. Nivolumab treatment of relapsed/refractory Epstein-Barr virus–associated hemophagocytic lymphohistiocytosis in adults. Blood. 2020;135:826–33. 31914172 10.1182/blood.2019003886

[9] Sawada A Inoue M Kawa K. How we treat chronic active Epstein-Barr virus infection. Int J Hematol. 2017;105:406–18. 28210942 10.1007/s12185-017-2192-6

[10] Bollard CM Cohen JI. How I treat T-cell chronic active Epstein-Barr virus disease. Blood. 2018;131:2899–905. 29712633 10.1182/blood-2018-03-785931 PMC6024635

[11] Song Y Wang J Wang Y. Ruxolitinib in patients with chronic active Epstein-Barr virus infection: a retrospective, single-center study. Front Pharmacol. 2021;12:710400. 34552486 10.3389/fphar.2021.710400 PMC8450490

[12] Xu N Fan HW Huang XM. [Clinical features of adult patients with chronic active Epstein-Barr virus infection]. Zhonghua Nei Ke Za Zhi. 2018;57:811–5. 30392236 10.3760/cma.j.issn.0578-1426.2018.11.004

[13] Luo YH Yang J Wei A. Haploidentical hematopoietic stem cell transplantation for pediatric patients with chronic active Epstein-Barr virus infection: a retrospective analysis of a single center. World J Pediatr. 2021;17:626–36. 34739695 10.1007/s12519-021-00470-9

[14] Matsui S Takeda Y Isshiki Y. [Chronic active Epstein-Barr virus infection with marked pericardial effusion successfully treated with allogeneic peripheral blood stem cell transplantation]. Rinsho Ketsueki. 2016;57:624–9. 27263789 10.11406/rinketsu.57.624

[15] Jelinek T Paiva B Hajek R. Update on PD-1/PD-L1 inhibitors in multiple myeloma. Front Immunol. 2018;9:2431. 30505301 10.3389/fimmu.2018.02431 PMC6250817

